# Targeting energy, nucleotide, and DNA synthesis in cancer

**DOI:** 10.3389/fonc.2025.1736064

**Published:** 2025-12-04

**Authors:** Ben Zion Vider

**Affiliations:** Independent Researcher, Moshav Herut, Israel

**Keywords:** cell cycle dominance, cell cycle related metabolism, nucleotides and DNA synthesis, cancer metabolism, anti-metabolites, triad combinations

## Abstract

Cancer represents a disease in which genetic alterations reassert the dominance of the cell cycle over all other cellular processes. From the earliest stages of evolution, the coupling of energy utilization with nucleotide and DNA synthesis established replication as the central driver of cellular behavior. In cancer, this evolutionary logic is replayed in reverse. Hyperactivation of the cell cycle drives hyperactivation of its metabolic core, while the loss or inactivation of tumor suppressor genes, many of which are cell-type specific, links this accelerated proliferation with altered cell fate. With virtually unlimited energy available, malignant cells amplify nucleotide production and DNA replication without restraint. This Perspective proposes that anti-metabolites, long-standing pillars of cancer therapy, can be redesigned to target the main components of DNA metabolism. By rationally combining these anti-metabolites into synergistic triads (three anti-metabolites, well selected, administered together), therapy may dismantle the metabolic foundations of cancer and achieve more durable control across tumor types. The combinations that could yield meaningful progress are outlined and discussed.

## Introduction

Cancer formation is a disease in which a genetic phenomenon reflects the increased dominance of the cell cycle (1). From the earliest stages of evolution, nucleotide production and DNA synthesis became the metabolic core that established replication as the central driver of cellular behavior. It began with ancient enzymes that coupled energy utilization with molecular synthesis. Among the molecules produced, DNA first emerged as a metabolic reservoir and later as the genetic code that instructed its own replication, creating a self-reinforcing loop. This process made the cell cycle the master regulator of life.

In cancer, this evolutionary logic is replayed in reverse. Hyperactivation of the cell cycle drives hyperactivation of its metabolic core. The loss or inactivation of tumor suppressor genes, many of which are cell-type specific, unites two fundamental processes: accelerated proliferation and altered cell fate (2). With virtually unlimited energy available, malignant cells amplify nucleotide production and DNA replication without restraint.

The therapeutic implication is clear: anti-metabolites can serve as central pillars of cancer therapy through rational strategies that dismantle the metabolic foundations of DNA replication. This approach may guide new research efforts targeting the three key components of this metabolic path, namely energy supply, nucleotide synthesis, and DNA synthesis, by designing novel anti-metabolites that inhibit these processes simultaneously and form a triad.

This Perspective outlines the conceptual and therapeutic roadmap for such an approach. It reviews the major metabolic stages and principal classes of anti-metabolites that target nucleotides, folate, and amino acids, while also addressing the potential benefit of supplementing specific metabolites in large quantities. Finally, it considers existing therapeutic combinations that approximate this triadic logic and discusses how refining them could lead to meaningful advances in anti-metabolite-based cancer therapy.

## Cell cycle genes and cell type specific genes

In multicellular organisms, cellular proteins can be broadly divided into two groups: those directly related to the cell cycle and those that define cell type. Each group includes both accelerators and inhibitors of proliferation. Most cell-cycle-related genes promote progression and, in a cancer context, function as oncogenes, whereas a minority act as tumor suppressors.

By contrast, many cell-type-specific genes tend to suppress the cell cycle, as their expression consumes time and energy and often produces adhesion molecules that inhibit proliferation and help maintain multicellular organization. Still, some encode receptors whose ligands stimulate cell-cycle entry (2).

Cell-cycle genes are largely shared across different cell types, and cell-type-specific genes, although more diverse, still display notable structural and functional overlap. This indicates that the dichotomy between the two groups is not absolute. Moreover, even cell-type-specific programs remain subordinate to the control of the cell cycle (3).

In summary, although cell-type-specific networks can restrain proliferation, everything a cell produces ultimately feeds back into the cell cycle.

## Evolutionary origins of the cell cycle

**Figure 1:**
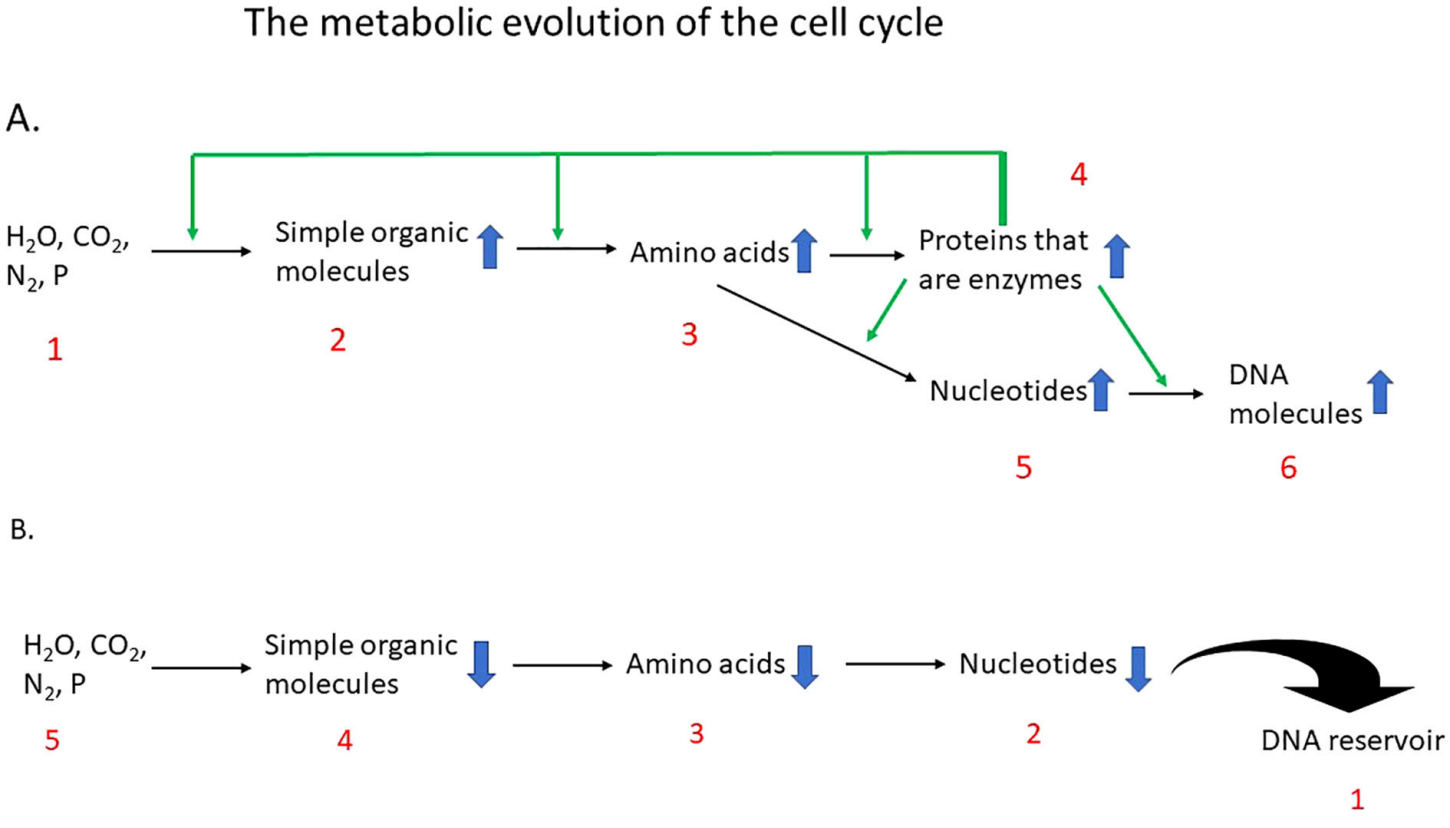
**(A)** During the establishment of cellular metabolic networks, the accumulation of reactants led to a proportional increase in product formation. In the schematic, entities 2, 3, 4, and 5 act both as reactants and products, whereas entity 1 functions solely as a reactant and entity 6 solely as a product. Black arrows denote biochemical reactions, and green arrows represent the enzymatic activities catalyzing these reactions. The rising concentration of each product (blue arrows) promotes forward reaction flux, creating a self-reinforcing dynamic. Entities 2, 3, and 4 constitute a positive feedback loop in which amino acids are converted into proteins that serve as enzymes, which in turn catalyze the synthesis of additional enzymes. **(B)** The emergence of DNA as the final product, an entity capable of generating virtually limitless copies of itself, acts as a driving force that pulls the preceding reactions forward by continuously depleting their products (as indicated by the blue arrows). Entities 1 through 5 illustrate the sequential progression of events that propel the reactions in this forward direction. The principles illustrated in **(A, B)** complement one another and together describe the metabolic logic underlying DNA synthesis.

Cells are composed of DNA and proteins, with RNA likely predating DNA in evolution (4). Their emergence depended on the energy stored in organic molecules and on the gradual appearance of primitive enzymes whose dual binding sites allowed the coupling of energy utilization with synthesis. In early evolution, amino acids generated enzymes, and enzymes in turn synthesized more amino acids and more proteins, establishing a self-sustaining cycle (**Figure 1A**).

As amino acid synthesis advanced, nucleotides began to accumulate, initially as energy carriers and later, through emerging polymerase activity, linking to form the DNA double helix (5) (**Figure 1A**). The rise of DNA marked a decisive turning point: reactions no longer depended solely on the availability of reactants. The final product, DNA, now served as a reservoir that drew pathway intermediates forward and accelerated biochemical flow (3) (**Figure 1B**).

Not all proteins produced during this stage functioned as enzymes; some served structural roles. Among enzymes, key nucleotide-binding proteins evolved gradually, beginning with ancient proteins containing ATP-binding pockets and ultimately giving rise to those capable of linking nucleotides together (6). DNA then assumed the role of genetic code, enabling the production of a far wider range of proteins. With this shift, coded proteins joined or replaced those that had previously assembled spontaneously (7).

The enzymatic network supporting nucleotide and DNA synthesis quickly became self-reinforcing, turning replication into the central event governing cellular timing and behavior (8).

In summary, three crucial developments defined this evolutionary transition. The first was the formation of enzymes, which made a self-sustaining loop that led to enzyme formation plausible. The second was the emergence of DNA, made possible by enzymes. The third was the moment when DNA began directing protein synthesis, establishing the cell cycle as a self-reinforcing process in which DNA instructed the production of molecules that, in turn, generated more DNA (9). Thus, the formation of the cell cycle, and indeed of the cell itself, was first and foremost a biochemical event and only later a genetic one.

## Cancer as a loss of restraint and metabolic overdrive

Once a cancerous mutation occurs, it shifts the balance toward cell-cycle progression. As the cell cycle becomes augmented, it begins to amplify itself: as opposing forces weaken, the activating forces grow stronger. Through mechanisms not yet fully understood, this self-reinforcing process promotes the accumulation of additional activating mutations in oncogenes and the inactivation of tumor suppressor genes, further accelerating malignant progression. The same evolutionary mechanism that once gave DNA synthesis primacy as the driver of cellular reactions reasserts its dominance in cancer (10).

What began in evolution as nucleotide production and DNA synthesis establishing the dominance of the cell cycle is replayed in cancer in reverse: hyperactivation of the cell cycle now drives hyperactivation of the metabolic routes at its core (11). There is a close connection, described in various review works, between specific well-known genetic mutations in genes like *RAS* and *MYC* and the activation of signaling pathways and the metabolic shifts that are typical of cancer (12, 13). Recognizing that metabolism is both a central driver of cancer formation and an accessible therapeutic target reinforces the rationale for directing cancer treatment toward anti-metabolites (10, 11).

## How loss of cell-cycle control is also a change in cell identity

Cancer is not solely a matter of unchecked replication; it also reflects a fundamental reprogramming of cellular behavior and identity. These two phenomena are closely linked, as tumor suppressor genes are largely cell-type specific, and their loss unites two defining hallmarks of malignancy: the loss of proliferative restraint and the alteration of cell fate.

In this way, loss of proliferative control and alteration of cell fate are two aspects of the same event. This is clearly seen in the epithelial-to-mesenchymal transition (EMT), a process in which epithelial cells lose polarity and adhesion. In doing so, they relinquish epithelial characteristics as well as cell-cycle inhibition, while acquiring mesenchymal traits and invasive capacity (14–16).

## Anti-metabolites: from origins to renewed potential

The use of anti-metabolites has accompanied cancer therapy since its earliest days. These agents have shown particularly impressive success in hematologic and childhood malignancies, though their impact has been more limited in adult and solid tumors (17).

Armed with a well-established understanding of the close connection, both in evolution and in cancer, between the cell cycle and nucleotide and DNA metabolism, this Perspective explores their still-underutilized potential.

An inspiring finding that highlights this potential comes from a clinical study showing that, although rare (about 1%), therapy of metastatic colon cancer with 5-fluorouracil (5-FU) alone can have a long-lasting impact, with responses extending beyond five years, including regression and even cure (18). This observation suggests that anti-metabolites may, under certain conditions, exert a profound therapeutic effect. It also demonstrates that drugs initially regarded as modest in efficacy can still achieve remarkable outcomes in specific biological contexts (11).

## Targeting the three pillars of DNA metabolism

Blocking energy supply, nucleotide synthesis, and DNA synthesis represents the guiding principle of anti-metabolite design and application in this Perspective. Anti-metabolites that block reactions related to each of these functions already exist in the arsenal of cancer therapy. Developing new ones, knowing their potential effect when combined together, might further enhance their impact. Combining anti-metabolites that interfere with these three steps in one protocol as a triad might increase their therapeutic effectiveness. It might also reduce toxicity due to the possible synergistic effect of these anti-metabolites, which may enable reducing the amount of each one.

## Current multi-agent strategies targeting DNA metabolism

Although at first glance the use of three anti-metabolites in a single regimen is rare, initial experiments of this kind have been attempted in the past (19, 20). Still, in recent years, combinations of two anti-metabolites, or their functional equivalents, are much more common in current protocols for major solid tumors than might initially appear.

What does the term “equivalent to anti-metabolites” mean? When reviewing the main therapeutic regimens used against prevalent cancers, many include one drug that targets and interferes with DNA synthesis, often not an anti-metabolite but still acting at the DNA-synthesis stage, and a second drug that is an anti-metabolite, primarily affecting nucleotide synthesis.

The drug that interferes with DNA synthesis but is not itself an anti-metabolite is what I regard as an anti-metabolite equivalent. It may also be described as anti-metabolite like.

Such protocols typically combine an anti-metabolite that blocks one of the steps in nucleotide synthesis, such as 5-fluorouracil (5-FU), pemetrexed, or gemcitabine (the latter also affecting DNA synthesis), with a drug that disrupts DNA synthesis but is not an anti-metabolite, belonging instead to the class of topoisomerase inhibitors, alkylating agents, or platinum-containing compounds. Together, these agents form the therapeutic backbone that targets two of the three fundamental stages of DNA metabolism. These regimens constitute cornerstone treatments for pancreatic cancer, colorectal cancer (CRC), non-small cell lung cancer (NSCLC), breast cancer, leukemias, and several other major cancers (17, 21).

## Current protocols approximating the triadic anti-metabolite therapy concept

A prominent example of a protocol that employs two anti-metabolites, in addition to agents that interfere with DNA synthesis as described earlier, is the regimen used for metastatic pancreatic ductal adenocarcinoma (PDAC). This protocol demonstrates superiority over other regimens, although it is still far from providing a true breakthrough in this challenging cancer. It involves sequential administration of nab-paclitaxel plus gemcitabine followed by modified FOLFOX-6 (oxaliplatin, leucovorin, and 5-FU) (nab-P/Gem-mFOLFOX), underscoring that more aggressive combination regimens in metastatic PDAC improve survival (22). The option to include other anti-metabolites as part of these combinations while replacing an existing one should not be excluded. For example, several *in vitro*, *in vivo*, and clinical protocols have been conducted over the years that examined the use of the antifolate drug pemetrexed with gemcitabine or after failure of gemcitabine treatment. The results of the clinical trials point to minimal advantage of pemetrexed, and possibly no advantage at all (23, 24). Still, the impressive *in vitro* effect of pemetrexed on PDAC cell lines and indications of its effectiveness in *in vivo* experiments are tempting reasons to try new combinations that include this anti-metabolite (25, 26). A wider *in vitro* approach looking for new anti-metabolite combinations might help improve the therapeutic outcome of this relatively unresponsive cancer.

In relapsed or refractory acute myeloid leukemia, two anti-metabolites, fludarabine and cytarabine, are used together with a topoisomerase II inhibitor that disrupts DNA synthesis, leading to clinical improvement in the course of the disease (27).

In the example of pancreatic cancer described above, the bioavailability of chemotherapeutic drugs is enhanced through the use of albumin-based nanoparticles that carry paclitaxel, thereby increasing drug uptake by pancreatic cancer cells while reducing systemic toxicity.

## Anti-metabolites that are nucleotides or nucleotide scaffolds

This group of anti-metabolites includes 5-fluorouracil (5-FU), gemcitabine, capecitabine, and several others. They act either by interfering with the final steps of nucleotide synthesis, by disrupting the conversion of ribonucleotides to deoxyribonucleotides, or, in some cases, by directly interrupting DNA synthesis.

5-fluorouracil (5-FU), as a single agent, has limited efficacy in colon cancer but becomes far more potent when combined with folinic acid. Folinic acid, a reduced derivative of folate, stabilizes the binding of 5-FU to thymidylate synthase by locking the enzyme into a conformation that enhances inhibition (28). This exemplifies the principle that many enzymes contain more than one binding site, allowing drug combinations to be designed to exploit cooperative stabilizing interactions. Folinic acid also prevents folate depletion that might otherwise occur during nucleotide synthesis (29, 30).

## Folate anti-metabolites

Folate contributes a carbon atom and hydrogens in several steps of nucleotide synthesis, including the conversion of uracil to thymidine, and is therefore essential for DNA replication.

Both methotrexate and the more potent pemetrexed act as anti-metabolites that interfere with this folate-dependent activity. The involvement of folate in multiple stages of nucleotide formation highlights its significant yet still underutilized potential in cancer therapy (31–33).

## Glutamine-directed anti-metabolites

Glutamine is a central molecular intermediate in many cell-cycle reactions, both in normal cells and, even more so, in cancer cells. Thus, anti-metabolites directed against glutamine have been designed and tested for many years in cancer therapy trials. Although toxicity halted most of these efforts, broader studies exploring alternative metabolites or lower concentrations should be pursued (34).

During nucleotide synthesis, glutamine donates its side-chain amide nitrogen in three reactions of purine biosynthesis and in one reaction of pyrimidine biosynthesis. In purine synthesis, glutamine-PRPP amidotransferase couples PRPP with glutamine, establishing the first committed step.

In terms of energy metabolism, glutamine replenishes the tricarboxylic acid (TCA) cycle with carbon skeletons, a phenomenon known as glutamine addiction, which is particularly pronounced in cancer cells (35, 36). Thus, glutamine anti-metabolites would serve both as central inhibitors of energy supply and as inhibitors of the various steps of nucleotide synthesis.

## Amino acid anti-metabolites beyond glutamine

Aspartic acid and asparagine share the same side-chain structure as glutamic acid and glutamine but are shorter by one carbon atom. Anti-metabolites directed against either asparagine or aspartic acid may hold significant therapeutic potential, as much of the aspartic acid molecule becomes incorporated into the pyrimidine ring during nucleotide biosynthesis.

In the first three steps of purine synthesis, glutamine, glycine, and formyl-tetrahydrofolate are all required. Notably, the entire serine molecule ultimately becomes integrated into the purine structure through these early reactions. Therefore, any anti-metabolite directed against glutamine, glycine, folate, and especially serine could exert strong inhibitory pressure on the early stages of nucleotide synthesis (33, 37, 38).

## Metabolic reorganization in cancer: amino acid distribution and therapeutic implications

In normal cells, amino acids are used primarily as building blocks for structural proteins, including those incorporated into the cell membrane and peripheral regions of the cytoplasm. In cancer cells, however, the situation is markedly different. With an increased nucleus-to-cytoplasm ratio and heightened activity of the cell cycle and nucleotide synthesis, amino acids become more centrally concentrated. This altered spatial organization may influence the activity of amino-acid-directed anti-metabolites, as higher nuclear concentrations in cancer cells could modify how these compounds interact with their molecular targets.

In this context, amino-acid anti-metabolites containing, for example, negatively charged residues such as carboxylate, phosphonate, or sulfonate groups may preferentially accumulate within the nuclear region. Their build-up could further disrupt nuclear organization in cancer cells and contribute to cytotoxicity, adding another layer of selective pressure against malignant growth.

## Future directions for anti-metabolite triads

In the previous sections, I discussed various avenues for the new design of anti-metabolites. When evaluating these new agents, initial testing should be performed *in vitro* in pairs with established drugs such as gemcitabine, 5-FU with folinic acid, or pemetrexed, at concentrations below their cytotoxic thresholds. Once a diverse set of new compounds is developed, a systematic program should be initiated to test rational three anti-metabolite combinations, a triad, *in vitro*.

Such triads might, for example, include gemcitabine and 5-FU with folinic acid in combination with a glutamine-directed anti-metabolite, or gemcitabine, a folate inhibitor, and a glutamine- or serine-directed anti-metabolite.

Today, large screening projects looking at the effects of many cancer drugs in various combinations on many cancer cell lines are possible (39). It becomes mainly a question of focusing on anti-metabolites and their systematic application in pairs and even in triplets across various cancer cell lines, which might reveal new effective combinations for different cancer types.

Still, the screening and clinical testing phases will likely be complex, as certain combinations of anti-metabolites might be assisted by the addition of a native metabolite, and until tested it will be hard to know which. This supplementary metabolite could enhance anti-metabolite activity in one of the following manners: by engaging a second enzyme-binding site, thereby improving drug activity, reducing resistance arising from cancer-cell metabolic plasticity, and lowering toxicity to normal tissues (38).

This orchestrated effort might be supported by identifying correlations between distinct genetic mutation patterns and the susceptibility of cancer cells to specific anti-metabolites and, likely, to particular anti-metabolite combinations. Such correlations have been found in several instances, such as between intact p53 in CRC and the effectiveness of 5-FU *in vitro* and in the clinic (40, 41). In a different setting, NSCLC cells with mutant KRAS demonstrate a better response to antifolates like pemetrexed than NSCLC cells with wild-type KRAS (12). Finding such genetic-to–anti-metabolite response connections might enable the application of personalized anti-metabolite combination therapy.

I would like to end this section by mentioning an example of a triad success. Though this example is outside the scope of cancer therapy, I believe it has relevance to it. It relates to the development of a combination of three antiviral drugs for HIV-1. The virus develops resistance to each drug alone within days or weeks of use, but the combination of three antiviral drugs together results in a very impressive therapeutic success and abolishes this resistance (42). In this way, a situation that was close to failure turned into an impressive therapeutic triumph.

## Conclusion

This Perspective presents an approach guided by past clinical experience and by the necessity to interrupt the three steps in metabolism that underpin cell-cycle dominance. Effective treatment of cancer will depend on the use of three anti-metabolites administered together, each acting at a distinct step within the overall process of DNA synthesis. It is conceivable that, within many existing drug combinations, the addition of a single innovative and effective anti-metabolite can tip the balance toward success.

The development of several such new anti-metabolites could result in a therapeutic breakthrough. Although the natural tendency to generate new mutations is an inherent feature of cancer progression, targeting the disease at its metabolic foundations may diminish the impact of such mutations on its course.

By providing a clear and coherent framework, this Perspective might increase the likelihood that meaningful advances in cancer therapy will arise from a renewed focus on metabolic logic and the strategic use of anti-metabolite triads.

## Data Availability

Publicly available datasets were analyzed in this study. This data can be found here: Data Availability Statement This article presents a theoretical and conceptual analysis and does not include original data. All information discussed is derived from previously published work cited in the References section.
